# Improving shared decision-making around antimicrobial-prescribing during the end-of-life period: a qualitative study of Veterans, their support caregivers and their providers

**DOI:** 10.1017/ash.2024.61

**Published:** 2024-05-17

**Authors:** Cassie Cunningham Goedken, Erin Balkenende, Daniel Livorsi, Karleen Giannitrapani, Matthew McCaa, Gosia Clore, Michihiko Goto, Alexandre R. Marra, Eli N. Perencevich

**Affiliations:** 1 Center for Access and Delivery Research and Evaluation (CADRE), Iowa City VA Health Care System, Iowa City, IA, USA; 2 University of Iowa, Carver College of Medicine, Iowa City, IA, USA; 3 Center for Innovation to Implementation (Ci2i), VA Palo Alto Health Care System, Menlo Park, CA, USA; 4 Primary Care and Population Health, Stanford University School of Medicine, Stanford, CA, USA; 5 Hospital Israelita Albert Einstein, São Paulo, Brazil

## Abstract

**Objective:**

Antimicrobials are frequently used for palliation during end-of-life care, but adverse effects, such as antimicrobial resistance, are a concern. Shared decision-making is beneficial in end-of-life care conversations to help align antimicrobial-prescribing with patient preferences. However, there is limited data regarding optimal incorporation of antimicrobial-prescribing discussions into shared decision-making conversations. We explored healthcare provider, patient, and support caregiver (eg, family member/friend) perceptions of barriers and facilitators to discussing antimicrobial-prescribing during the end-of-life period.

**Design:**

Qualitative study.

**Participants:**

Healthcare providers; palliative care/hospice care patients/caregivers.

**Methods:**

We conducted semi-structured interviews on shared attitudes/beliefs about antimicrobial-prescribing during end-of-life patient care at one acute-care and one long-term-care facility. Interviews were analyzed for thematic content.

**Results:**

Fifteen providers and 13 patients/caregivers completed interviews. Providers recognized the potential benefit of leveraging shared decision-making to guide antimicrobial-prescribing decisions. Barriers included limited face-to-face time with the patient and uncertainty of end-of-life prognosis. Patients/caregivers cited trust, comprehension, and feeling heard as important characteristics which act as facilitators in fostering effective shared decision-making around antimicrobial use. Communication in which providers ensure patients are involved in shared decision-making discussions could be increased to ensure patients and their providers develop a mutually agreeable care plan.

**Conclusions:**

Shared decision-making is a practice that can guide antimicrobial-prescribing decisions during end-of-life care, thus potentially minimizing antimicrobial-related adverse effects. Our findings highlight opportunities for increased shared decision-making around antimicrobial use during end-of-life care. Interventions designed to address the identified barriers to shared decision-making have the potential to improve antimicrobial-prescribing practices at end-of-life.

## Introduction

Antimicrobial resistance is a growing public health crisis that is caused, in part, by unnecessary antimicrobial-prescribing. The overuse of antimicrobial therapy has been well identified as a concern across the spectrum of health care, including in palliative care and hospice care settings.^1–3^ In a study by Albrecht et al., during the last seven days of life, 27% of patients received at least one antimicrobial and 1.3% received three or more antimicrobials.^4^ More recently, Clark et al., identified that only 42% of hospice patients receiving antimicrobials were prescribed antimicrobials appropriately.^5^

There are several barriers to improving antimicrobial-prescribing in palliative care and hospice care settings. Antimicrobials are frequently used empirically for end-of-life patients, as confirmatory laboratory tests can be viewed as burdensome for the patient. In addition, patients’ impaired cognition may make it difficult to assess for infection symptoms. Further, prescribers often mistakenly believe the possible symptom relief provided by prescribing antimicrobials for a suspected infection outweigh the potential antimicrobial-related harms for end-of-life patients.^6^ However, adverse effects are linked with antimicrobial use, including, *Clostridioides difficile* infection and the emergence of antimicrobial resistance.^3,7–9^

Shared decision-making, which denotes the exchange of information between the patient, their caregiver, and their healthcare provider, is beneficial in end-of-life care conversations to help align medical care with patient preferences. The decision to initiate antimicrobials during end-of-life care is complex.^4,6,8^ It has been well documented in the literature that practice recommendations for antimicrobial-prescribing at end-of-life should involve shared decision-making.^3,10^ Specifically, joint discussions between healthcare providers and the patient/support caregiver regarding antimicrobial-prescribing, including their potential risks and benefits, as well as understanding the patient’s goals are key.^11^ However, there is limited data on barriers and facilitators to incorporating antimicrobial-prescribing discussions into shared decision-making conversations.

We explored provider, patient, and support caregiver (eg, family member or friend) perceptions of barriers and facilitators to discussing antimicrobial-prescribing during the end-of-life period. Identification of potential barriers and facilitators to shared decision-making during the end-of-life period may guide antimicrobial-prescribing discussions.

## Methods

### Study design

We used an ethnographic approach for epistemological reasons to better understand barriers and facilitators to antimicrobial-prescribing decisions during end-of-life.^12,13^

### Setting and participants

Purposive and convenience sampling strategies were used to identify semi-structured interview participants. The Veterans Health Administration is the largest integrated healthcare system in the United States, providing care to over 9 million patients.^14^ Semi-structured interviews with healthcare providers and patients/support caregivers were conducted at two Midwest Veterans Affairs facilities, one acute care and one long-term care (ie, community living center) from November 2020 to May 2022.

### Definitions

We defined hospice care as caring for a patient during the last 6 months of life and palliative care as caring for a patient after the diagnosis of a serious illness.

### Data collection

Healthcare providers were invited to participate via e-mail and interviewed via telephone or video conference (CCG and EB). Patients/support caregivers were identified by hospital admission data. Patients who recently received a hospice care or palliative care consultation were approached and interviewed in-person (CCG and EB). Interview guides were developed by the research team with questions focusing on attitudes and beliefs about antimicrobial-prescribing during end-of-life care.

Interviews were audio-recorded and transcribed verbatim, lasting an average of 39:01 (healthcare provider) and 15:21 (patient/caregiver) minutes. All participation was voluntary. One patient/caregiver interview was not recorded, due to participant refusal; detailed notes were taken and used for analysis. Data collection ended when thematic saturation was reached.^15^

### Ethical considerations

The study was approved by the Institutional Review Board at the University of Iowa (#: 201909834) and the Research and Development Committee at the Iowa City VA Health Care System. Verbal consent was provided prior to interviews.

### Data analysis

Transcripts were analyzed using MAXQDA, a qualitative data program (VERBI Software, Berlin, Germany). Thematic content analysis^16,17^ was performed by an interdisciplinary team which included trained social scientists (CCG, EB, KG, and MM) and infectious disease clinicians (EP and DL). First, our analysis process involved reviewing three transcripts by the large group (CCG, EB, KG, MM, EP, and DL) and a codebook was developed based on a priori research questions and emergent content.^18^ Eleven percent of the transcripts were coded via group consensus (CCG, EB, KG, MM, EP, and DL), a process that involved all team members coding transcripts prior to meetings where the final coding consensus was entered into MAXQDA during group discussion. The remaining 89% were coded by paired members of the analysis team (EB, MM, CCG). Paired consensus coding meetings started after the codebook was developed. Discrepancies were resolved by the larger team.

Next, we subcoded one of the most frequently applied codes “shared decision-making.” We consensus subcoded all text segments either in the large group or paired group setting into “goals of care” and “quality of life.”

## Results

Fifteen prescribing healthcare providers and 13 patients/support caregivers who were currently receiving hospice care or palliative care treatment were interviewed regarding attitudes/beliefs about antimicrobial-prescribing and experiences during end-of-life care. Of the thirteen interviews, 62% were patients and 38% were support caregivers. Prescribing healthcare provider (47% female; 53% male) roles included physicians (*N* = 13; 40% serving in a leadership role) and pharmacists (*N* = 2), with an average of 4.53 years (SD:2.89) in their current role. Most patients (70%) were receiving palliative care, all were male, and average age was 73.9 years (SD:8.02). Three overarching themes emerged. Representative quotations can be found in Table 1.

**Table 1.** Quotations of key themes from semi-structured interviews with healthcare providers, patients, and/or support caregivers

Theme 1: Healthcare providers highlighted that shared decision-making with the patient and support caregivers was key for guiding optimal antimicrobial treatment	
Provider ID#06, Site 1	*(…) It’s really assessing the goals of care, in the stage of end-of-life. (…) it may be appropriate if the goal is to prolong life and the patient’s underlying disease is not as bad. But it’s likely to be inappropriate if the disease is advanced, you know, like in a terminal patient or hospice patient. IV antibiotics at this point are futile. (…) to my knowledge [no] data’s been shown to support significant symptomatic relief. Then I think there might be situations where there may be symptomatic relief but ultimately, that decision making, you know, the goals of care and what stage of the end-of-life are they in? In addition to, you know, risk versus benefits, evidence-based medicine, (…) I think it’s such an important thing to consider.*
Provider ID#11, Site 1	*More in the hospice setting, ideally at least, they would be having that conversation and having a more fruitful conversation with the patient about “Is this in line with what you’re looking for?” And I think in hospice in some settings, it would make sense to give antibiotics if they are in there for cancer, heart failure, and they have a urinary tract infection that may make a lot of sense, because it may make them more comfortable, decrease their symptom burden by treating that infection. The relative risk of the antibiotics are relatively small at that point.(…), you really have to let the patient’s preferences lead that treatment decision.*
Provider ID#02, Site 1	*(…) a lot of the decision making is not being driven so much by diagnostic cut and dry, but it’s also being driven by conversations with the family, conversations with the patient about what their preferences are in terms of care, so it’s almost an entirely different realm of making medical decisions than what we often deal with in patients who are under hospice.*
Provider ID#01, Site 1	*(…) and so, it’s important to- it’s great when the family has explicit information. When they don’t, they have to sort of gather, um, values that they- that their loved one had. But the assumption, uh, the importance of having the family involved is that there is an assumption among physicians that every infection should be treated in every situation. And, um, and that’s just how we’re taught. So it-it’s important to do shared decision making in the palliative care patient who has a serious illness. Um, in part to make sure that people understand that they have an exit strategy.*
Provider ID#14, Site 2	*I think it’s [antimicrobial prescribing] somewhat dependent on the underlying condition, as well as what the patient’s goals are. If they’re looking for a particular, you know- to reach a certain goal, like go to their child’s wedding or something like that, and, you know, they get an infection in the interim and you think you can get them over the hump to be able to, you know, do that life goal, then that’s one situation versus, you know, if they’re- if they’re kind of more ready to pass. (…) I think it’s definitely a lot more complicated kind of decision making than it would be for somebody with standard care. Because most times in standard care an infection is a reversible condition. (…) in many cases, somebody who’s on hospice, it would still be appropriate to try to treat reversible conditions and most people would want that. But again, if they’re, you know, very close to death and, you’re not sure if it’s gonna be fruitful for them or help them meet a goal, then that might be a different situation.*
Provider ID#03, Site 1	*(…) I think the biggest thing is (…) talking to patients about their goals and their values, and, you know, kind of what’s most important to them. As well as then asking them to, you know, talk to their family about their goals, values, and wishes, and then ultimately getting those things in writing.*
Provider ID#01, Site 1	*(…) if I were gonna draw a, you know, rectangle, I’d put a hypotenuse through the middle and say that, at the beginning of a serious illness it’s probably gonna look much like standard care. I don’t think you should get any passes for starting antibiotics empirically, ever. But as time goes on, goals are more paramount than clinical situations. And there needs to be more and more shared decision making with, patients’ families, and including goals and values. (…) I think it’s a continuum.*

Theme 2. Barriers to performing shared decision-making in regard to antimicrobials include time constraints and uncertainty of EOL prognosis	
Provider ID#03, Site 1	*(…) somebody has to ultimately make a decision and prescribe or not prescribe antibiotics. (…) it’s not always a clear-cut thing and (…) talking as a team and involving people is often ideal, and then there’s just the reality of time pressure and communication (…).*
Provider ID#01, Site 1	*(…) Most deaths in America are-are completely predictable. Cancer, heart disease, lung disease, Parkinson’s Disease. We know when people are going into, the final days, and it’s our job as physicians to give anticipatory guidance. That is, let them know, let patients and their families know what’s coming next. We are woefully inadequate in this process. Which is why people often land in the ICU because discussions about processes happen- happened ahead of time, and people who are dying (…) don’t have what I call, prognostic awareness to know, that that’s what’s happening (…).*
Provider ID#15, Site 2	*(…) the cumulative effect I would say of all of those chemotherapeutic regimens, radiation aside, provided, and surgery with, you know, devices such as ports inserted can lead to infections that are very difficult to control. And at that point, (…) both the patient, their family, and providers are frustrated that the end goal cannot be changed, (…) And as a way to kind of possibly look at it, and saying, “We’re gonna use antibiotics to kind of help tamp down this infection that is there, —proven (…) suspected,” And so, that’s usually the timeframe I get pulled into. A lot of times I think by explaining that “This is an end point that cannot be, unfortunately, changed, and antibiotics or antimicrobials are not gonna make a difference in the long-term.” I mean you’re supposed to convince the family and the patient to look at goals that can be achieved which is more comfort-driven as opposed to saying, “I’m gonna put a new port in. I’m gonna do six weeks of antibiotics. I’m gonna see how this plays out.”*
Provider ID#14, Site 2	*It [antimicrobial prescribing hospice vs. standard care] depends on how close they are to death. (…) one that may affect, you know, kind of your thought process about what the benefits of the antibiotic use could be, as well as (…) what kind of condition has qualified them for hospice. And whether that condition, like, the natural progression of that condition is an infection. So for example, when I talk to people about, you know, dying from cancer, some cancers maybe more likely to cause an infection, and that’s how most people die. (…) a big lung cancer that’s blocking off part of their lung is gonna, you know, more than likely cause an pneumonia at some point.*
Provider ID#06, Site 1	*Often antimicrobial stewardship principle application of, you know, evidence-based medicine. And even my impression is risk versus benefits don’t always apply to the palliative patient like it does in the general population. I think there’s often situations where, there’s decisions-, they’re driven by family. There’s decisions that the provider may make, using antibiotics that may-, might not be standard of care.*

Theme 3. Patient/support caregiver comprehension and trust help facilitate effective antimicrobial shared decision-making	
Caregiver#01, Site 1	*The palliative care doctor has been in to see me almost everyday that we’ve been here. So, yeah he’s been very involved in talking to me, (…) explaining things, and, you know, and-and again I had to just remind him that it’s- we talked to Dad and Dad’s position in life. Whatever he wants to do. (…) but then we talked to him, it’s his life, you know. Just supporting what he wants and not taking control and changing it, you know?*
Patient#03, Site 1	*Well, I’ve-, I’d say they [healthcare providers] talk to me. I’ve been able to get information about how I’m doing tests and so forth. Nobody is (…) not letting me know what was going on if I asked.*
Caregiver#05, Site 1	*Yeah, there’s been good, clear communication with that [healthcare providers discussing medications]. Because it’s- ups and changes the level of care we’re working with now is different than when he was there [non-VA facility], so it’s uh, yeah-, and the whole time it’s pretty well spelled out.*

Theme 1: Healthcare providers highlighted that shared decision-making with the patient and support caregivers was key for guiding optimal antimicrobial treatment.

“Goals of care” was defined as any discussion with the care team, patient, or support caregiver around the patient’s treatment plan, including impact on quality of life:

*Your goals of care conversation is basically a blueprint or a map that you make with the patient that helps steer you during this course all the way to end-of-life. [Provider ID#13, Site 2]*

To advise appropriately, providers need to understand the goals of the patient:

*(…) what we discussed (…) all stems from that conversation about what is important to the Veteran. (…) what makes life meaningful for them. And then, that helps you advise them. Like, “Here’s what I think as a physician, you know, would be the next best step in terms of your care.” Which is still shared decision making, it just starts with a conversation about what’s important to them. [Provider ID#09, Site 1]*

*Yeah, they [providers] were trying to involve the plan of attack here. Finally decided that there is cancer on my liver. And they’re not sure how they’re gonna do, -treat it, the cancer. Coming up with options all the time, we discuss those options. [Patient ID#08, Site 1]*

In some situations, the goal of a patient may be to prolong life to a desired timepoint, and this can influence the decision to prescribe antimicrobials:

*(…) But if you have [end-stage] COPD and your son’s graduating from school in six weeks and their goal is to make it to there. In that patient we might want to prescribe an antibiotic [to treat pneumonia] in hopes of extending their life to reach their goal and after their son graduates, then usually they can just say, “Okay, I don’t need antibiotics because I know I’m gonna die of COPD and probably will have pneumonia, there’s a very high chance for it, and as it gets worse, it’s probably not gonna extend my life or increase my quality of life,” so we would choose not to do antibiotics. [Provider ID#13, Site 2]*

In general, providers highlighted the need to recognize and shift their thinking away from treating infections and potential role for shared decision-making to help in doing so in the end-of-life setting:

*There’s so many different factors that come into play with antibiotics, especially when people are trying to shift gears from “we always treat, we want everything” as far as treating every infection to recognizing that many infections are end-of-life events in terminal patients. [Provider ID#10, Site 2]*

In 2017, the VA initiated a nationwide quality improvement project titled, Life-Sustaining Treatment Decisions Initiative (LSTDI) to promote personalized, patient-driven care for Veterans with serious illness by identifying, documenting, and honoring their goals of care preferences.^19^ The LSTDI is one mechanism to help guide the end-of-life shared decision-making process, as it was developed for primary care providers, or clinicians with the best relationship with the patient, to help ensure they have detailed goals of care conversations with patients. However, the life-sustaining treatment form can be challenging to navigate, when the different treatment options are presented like a menu of options rather than the life-sustaining treatment facilitating a larger discussion about what matters most to the Veteran:

*(…) I’ve participated in discussions where it (…) sounds like you’re providing the family with a menu. (…) “Do you guys want antibiotics?”(…). [ProviderID#08, Site1]*

Among the therapy options offered on the life-sustaining treatment form, antimicrobials are somewhat unique, as they are a function of standard medical care as illustrated in the following quotation:

*(…) you absolutely have to talk to the patient themself and their family, if they have family, and they’re willing to include the family. (…) because again antibiotics are something that in standard medical care and the culture in general, are kind of an expectation. You know if there’s a bacterial infection then you treat it with, an antibiotic. So, to not do that and to not discuss, that possibility, that option to not use antibiotics in certain cases, in the hospice, I think many people would probably feel, like something really important wasn’t talked about. And the flipside of that is, you know, some people may not understand there is an option not to use antibiotics in certain cases. And they might choose that option if they knew that because antibiotics do come with risks. [Provider ID#14, Site 2]*

The decision on whether to shift from prescribing to not prescribing antimicrobials relies heavily on end-of-life prognosis and the goals of care conversations providers have with their patients. In some situations, antimicrobials may merely prolong a patient’s suffering, which could motivate a provider not to prescribe: *“if the patient is dying right now, (…) I’m not going to give antibiotics. It’s going to prolong suffering.” [Provider ID#4, Site 1]*

Theme 2. Barriers to performing shared decision-making regarding antimicrobials include time constraints and uncertainty of end-of-life prognosis.

End-of-life goals of care conversations take time, as illustrated in the following quotations:

*(…) we just don’t do this [end-of-life conversations] nearly enough. And there’s a lot of barriers, I think the biggest barrier the physicians always speak of is time. It does take time to have those discussions. [Provider ID#03, Site1]*

An added barrier to having end-of-life conversations is the difficulty in providing the patient with an accurate prognosis of how much longer they have to live:

*there’s a cultural barrier, which we won’t be able to change very quickly. (…) whoever is working with the patient long-term, as they’re aging, as they’re getting sicker, if it’s just something that we can remember to offer them as an alternative. (…) it’s hard, because you don’t want to offer something as an alternative until you’re really sure they’re, you know, at the end-of-life. [Provider ID#14, Site 2]*

Highlighting this difficulty in prognostication, one provider recalled several patients who lived far longer than the medical team had predicted:

*And the CLC [community living center] hospice unit was designed really to be for those that are very end-of-life, that need aggressive symptom management. Now, having said that, we had a number of Veterans, actually several, that lived over a year. Some that you think have only a few weeks that end up living a number of months. It sometimes becomes really challenging to look at what are goals of treatment. [Provider ID#10, Site 2]*

Theme 3. Patient comprehension and trust help facilitate effective antimicrobial shared decision-making.

Patient/support caregiver responses predominately aligned with theme 3. From a patient perspective, important characteristics fostering effective shared decision-making include trust and closed-loop communication. Communication in which providers ensure patients are involved in the discussions and feel they’ve been heard, as well as to ensure they understand and comprehend the agreed upon care plan as illustrated by Patient ID#08, Site 1:

*Well, they made sure I understood and asked me if I had any questions, and if I did, they did their best to answer them.*

This patient went on to describe comprehension as:

*Yeah, they [healthcare team] were trying to involve the plan of attack here. Finally decided that there is cancer on my liver. And they’re not sure how they’re gonna do, treat it, the cancer. Coming up with options all the time, we discuss those options. What can and can’t go wrong with how it affects you and all that good stuff.*

Trust was often cited as an important component of shared decision-making. Involving the patient/support caregiver in their care discussions is a crucial part of the trust-building:

*You have to trust somebody, you know? And if you’ve got a doctor that’s open with you that you can trust, you’re in good hands. If you don’t trust your doctor, you’re gonna worry yourself to death. So if you trust your doctor, and you know, you’ll get much better care out of it. [Patient ID#10, SITE 1]*

## Discussion

### Main findings

Interview responses indicated healthcare providers recognize the potential benefit of leveraging shared decision-making and having a clear “blueprint” of the patients’ goals and wishes to guide antimicrobial-prescribing decisions; however, barriers included limited face-to-face time with the patient and uncertainty about the patient’s prognosis. Facilitators identified by patients and support caregivers largely centered on trust, comprehension, and feeling heard as important characteristics for effective shared decision-making.

Shared decision-making is considered a recommended practice for making antimicrobial-prescribing decisions during the end-of-life.^3,10^ Discussions surrounding patients’ goals of care can create opportunities for patients to make informed decisions about and contribute to their care, which has potential to alleviate provider challenges, as well as improve build patient trust.

Our findings identify healthcare provider and patient/support caregiver barriers and facilitators around shared decision-making regarding antimicrobial-prescribing at end-of-life. Previous studies have identified similar barriers to shared decision-making at end-of-life (eg, time constraints and prognosis uncertainty).^6,20^ Other barriers identified in published studies include providers’ discomfort with having these difficult conversations with patients,^6,21^ as well as residents’ lack of training, experience, and communication skills to conduct these nuanced end-of-life conversations.^22^

Future research should include a broader range of healthcare settings, incorporate quantitative data to complement qualitative findings and explore the perspectives of various healthcare providers and delve deeper into the specific factors influencing antimicrobial-prescribing decisions in end-of-life care. Further, we can enhance our understanding of the complexities surrounding shared decision-making and antimicrobial use and develop targeted interventions to improve uptake of shared decision-making into antimicrobial-prescribing decisions during end-of-life care.

### Strengths and limitations

Strengths of our study include in-depth qualitative interviews with healthcare providers as well as in-person patient/support caregiver interviews. A limitation is that our study was restricted to the Veteran Affairs Healthcare system. This could limit the generalizability of the results to other healthcare settings. The Veteran Affairs system may have unique characteristics, such as a specific patient population or organizational culture, that could impact the findings. Further, all patient participants were male. Antimicrobial use was not a qualitative interview recruiting strategy; thus, use was unknown for interview participants. Patient participants were hospitalized and very ill; thus, the study team sometimes had difficulty eliciting patient reflections.

## Conclusions

Palliative and hospice care settings have unique challenges that can make it difficult to ensure guideline-directed antimicrobial-prescribing. Improving antimicrobial-prescribing at end-of-life is a priority to minimize antimicrobial-related adverse effects. Our findings highlight an opportunity for greater use of shared decision-making when discussing antimicrobial use during end-of-life care. Interventions should be designed to address the identified barriers to shared decision-making.
